# Objective 3D Printed Surface Quality Assessment Based on Entropy of Depth Maps

**DOI:** 10.3390/e21010097

**Published:** 2019-01-21

**Authors:** Jarosław Fastowicz, Marek Grudziński, Mateusz Tecław, Krzysztof Okarma

**Affiliations:** 1 Faculty of Electrical Engineering, West Pomeranian University of Technology, Szczecin, 70-313 Szczecin, Poland; 2 Faculty of Mechanical Engineering and Mechatronics, West Pomeranian University of Technology, Szczecin, 70-310 Szczecin, Poland

**Keywords:** additive manufacturing, 3D prints, 3D scanning, image entropy, depth maps, surface quality assessment, machine vision, image analysis

## Abstract

A rapid development and growing popularity of additive manufacturing technology leads to new challenging tasks allowing not only a reliable monitoring of the progress of the 3D printing process but also the quality of the printed objects. The automatic objective assessment of the surface quality of the 3D printed objects proposed in the paper, which is based on the analysis of depth maps, allows for determining the quality of surfaces during printing for the devices equipped with the built-in 3D scanners. In the case of detected low quality, some corrections can be made or the printing process may be aborted to save the filament, time and energy. The application of the entropy analysis of the 3D scans allows evaluating the surface regularity independently on the color of the filament in contrast to many other possible methods based on the analysis of visible light images. The results obtained using the proposed approach are encouraging and further combination of the proposed approach with camera-based methods might be possible as well.

## 1. Introduction

One of the most dynamically growing technologies in the era of Industry 4.0 is undoubtedly the additive manufacturing widely known as 3D printing. The applicability of these technologies covers many areas of modern technology, industry, medicine, clothing, preservation of cultural heritage and even food production. The great variety of affordable 3D printers, which may be assembled at home with open source software, causes growing popularity of the cheapest devices utilizing the Fused Deposition Modeling (FDM) technology. Their principle of operation (initially preserved by the patent expired in 2009) is based on the melting process of plastic material (filament) and moving the extruder according to the defined tool path. The dripping filament hardens immediately and forms the visible layers which should adhere to each other. Some other solutions, however much less popular and more expensive, include selective laser sintering, stereolithography and inkjet printing.

The most typical materials used in FDM printing are Polyactic Acid (PLA) and Acrylonitrile Butadiene Styrene (ABS). Both types of filaments have different properties and the most popular thermoplastic polymer for low-cost 3D printers is the PLA filament. Although it is translucent in its natural form, it may be dyed using various colors. This fully biodegradable material can also be used for food packaging purposes but is less durable, more fragile and sensitive to heat in comparison to ABS. Therefore, its melting point is lower (around 150 ${}^{\circ }$C) as the ABS filaments require typically about 200 ${}^{\circ }$C. ABS filaments are more abrasion resistant and lightweight. They can be used to create low cost medical prostheses and has good mechanical properties. However, there are some concerns related to its potential toxicity, especially regarding fumes emitted during printing [1,2].

The final quality of 3D printed objects depends on various factors. Some of them are related to the quality of materials from which the printing device is made as well as the accuracy of its construction. The other elements influencing the quality of 3D prints might be the quality of the filament, temperature and other environmental conditions as well as improper printing parameters (e.g., filament’s delivery speed, wrong configuration of the stepper motors, inappropriate melting temperature for the specified type of filament, etc.). The most typical surface distortions may be related to under-filling (dry printing), over-filling or the presence of cracks without adequate adhesion of layers.

One of the future challenges is related to the detection of the internal imperfections of 3D printed objects. The application of X-ray tomography and ultrasonic imaging for the detection of embedded defects and altered printing orientation was examined by Zeltmann [3]. The presence of some internal distortions may also be detected using electromagnetic methods, e.g., terahertz non-destructive testing [4]. Nevertheless, the applicability of these technologies for on-line printing monitoring is very limited in opposition to cameras and reasonably small 3D scanners, which are mounted in some 3D printing devices. Therefore, in this paper, we focus on the assessment of the outer surfaces based on the regularity of visible layer patterns produced by the FDM printers.

Since the process of additive manufacturing is usually time-consuming and the production of a complicated object may take several hours or more, an obviously desired solution is the on-line monitoring of the progress of 3D printing. In such systems, the detection of low printing quality may lead to aborting the printing to save the filament, time and energy or—for smaller imperfections—performing some corrections for previously manufactured layers. However, such a decision is often dependent on hardware capabilities [5].

Some early ideas of the visual monitoring of the 3D printing progress have been related to the use of process signatures applied for fused deposition of ceramics [6,7] and detection of places with missing filament by monitoring the top surface of the manufactured object based on the process dynamics models [8]. Some predefined types of distortions, such as part jams and feeder jams, may be detected using the method proposed by Szkilnyk [9], although this approach is devoted mainly to machine vision fault detection of automated assembly machines. Similar methods for fault detection, which make use of Gaussian Mixed Models (GMMs), blob analysis, optical flow and running average, were presented recently by Chauhan and Surgenor [10,11].

Another exemplary defect detection system for plastic products, namely anaesthetic respiratory masks, based on computer vision, is presented in [12]. Recently, an interesting approach to optimization of tool paths during the 3D printing process was suggested by Fok et al. [13]. Not only does it save the filament and avoid the presence of visible strings, but it also significantly improves the visual quality of manufactured products. The additional advantage of this approach is the minimization of the time spent on traversing transitions.

Some interesting works related to 3D printing issues were published by Jeremy Straub, including the first machine vision system making it possible to detect of the lack of filament, based on five cameras and Raspberry Pi units [14]. Unfortunately, the proposed solution is very sensitive to camera motions as well as the changes of lighting conditions and additionally requires many stops during the printing process. Some other related papers describe alignment issues [15], cybersecurity problems [16,17] and human error prevention [18].

Some other recent attempts to the monitoring of the 3D printing process include the use of neural networks for the 3D inkjet printing of the electronic products [19], matching the reference images of the models of the 3D parts with their manufactured equivalents [20] and the 3D image correlation comparison with the CAD model using two cameras [21].

A recent paper written by Scime [22] presents the system for in-situ monitoring and analysis of powder bed images for the laser powder bed fusion machine. Nevertheless, some consequential anomalies that may be detected require previous training using the machine learning algorithms. As stated by the authors, the analysis of each layer requires approximately 4 s on a single 4.00 GHz i7-4770 processor, thus the method is relatively slow. Another application of machine learning for quality monitoring of 3D prints, based on the trained SVM model, was presented recently by Delli and Chang [23]. The main limitations of this approach are related to the use of top view camera and necessary stops of the printing process for image acquisition during manufacturing.

One of the successful attempts to the use of 3D scanning for multi-material 3D printing monitoring has been presented in the MultiFab platform [24]. The applied 3D scanner module is based on Michelson’s interferometer and full-field optical coherence tomography (OCT). Since the authors of the MultiFab system assume the lack of spatial details and significant textures, the direct application of structured light scanning is not possible. Nevertheless, for the low cost FDM based 3D printers, the visibility of each layer of the filament can be assumed for the side view cameras and the applicability of 3D scanners is worth investigating. To verify the usefulness of structured light 3D scanners, which have been successfully applied in many other applications such as CNC machines [25] and visual control of the loading cranes [26], some experiments were conducted for a dataset of several 3D printed flat surfaces subjected to 3D scanning followed by conversion of the obtained STL models into depth maps.

Since the main motivation of our research was the development of an objective method useful for automatic evaluation of 3D printed surfaces, one of the contributions of the paper is the verification of the usefulness of depth maps for this purpose. The novelty of the proposed approach is related to the use of the 3D scanning technology, providing the depth information, together with the development of the original entropy based surface quality assessment method, to increase the color independence of the obtained experimental results.

The rest of the paper consists of the short overview of the previous attempts to automatic quality assessment of the 3D printed surfaces, description of the proposed approach, presentation and discussion of obtained results and conclusions.

## 2. Methods of Quality Evaluation of 3D Printed Surfaces

Considering the assumptions related to the visibility of texture patterns caused by the use of low cost FDM devices and the application of the side view camera, the first approach to automatic quality assessment of 3D printed surfaces has been the use of texture analysis methods. For this purpose, a method based on the calculation of Haralick features determined from the Gray-Level Co-occurrence Matrix (GLCM) has been used [27]. Since the GLCM, also called the second-order histogram, represents the statistical information related to the neighbouring spatial relations between individual greyscale levels, some regularities may be expected, especially considering the vertical neighbourhoods. In fact, the periodicity of some features of the GLCMs calculated on the assumption that various offsets between the analyzed pixels may be observed. Further experiments made it possible to distinguish the scanned images from their equivalents captured by a camera and properly classify them into high and low quality samples with the use of homogeneity, independently on the image type [28]. Nevertheless, the application of this method requires many computations due to the necessity of analyzing tens of GLCMs and the obtained results have been verified for a limited number of PLA samples.

Another possible approach is based on the application of some image quality assessment (IQA) methods [29]. As the direct application of the most widely known full-reference IQA metrics, e.g., Structural Similarity (SSIM) proposed by Wang and Bovik [30], is not possible due to the lack of reference images, the self-similarity of the 3D printed surfaces is utilized. After the division of images into fragments, the local mutual similarity indexes are calculated to detect the low quality samples due to the presence of high local differences. Nevertheless, the method requires the additional matching of image fragments further examined together with the application of the Monte Carlo method to speed up the computations [31].

The first attempt to the use of image entropy for the assessment of the 3D printed flat surfaces is presented in a conference paper [32], where the direct application of image entropy is shown to be an appropriate method for the classification of high and low quality samples for a specified color of the filament. The method assumes that a perfect surface of the 3D printed sample observed from a side view camera should have only regular visible line patterns representing individual layers. For such regular patterns, the image entropy should be significantly lower than the values obtained for lower quality samples contaminated by various distortions. Nevertheless, the values obtained for the whole images, as well as the local entropy values calculated for the fragments of images, were strongly dependent on the color of the filament and the color to greyscale conversion. Therefore, to overcome this limitation, further experiments described in the present paper were made for the depth maps obtained from the 3D scans of the printed samples.

## 3. Proposed Method and Its Experimental Verification

**Figure 1:**
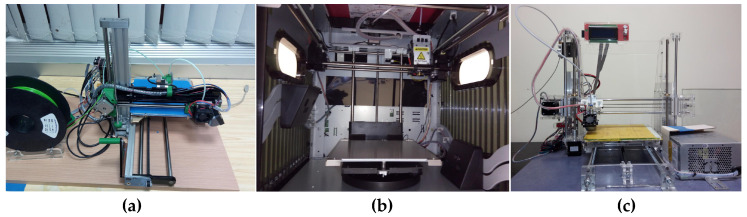
Three devices used for preparation of samples used in experiments: (**a**) RepRap Pro Ormerod 2; (**b**) da Vinci 1.0 Pro 3-in-1 (view of inner parts); and (**c**) Prusa i3.

To propose a reliable method of objective surface quality assessment, which would be independent on filament’s color and further verify its prediction accuracy, several dozen flat samples were printed using three different devices illustrated in Figure 1. Most were obtained using various colors of ABS filaments (108), however 18 of them were manufactured using various PLA materials. Most of the high quality prints were obtained using da Vinci Pro device and the same or very similar settings were also used for the other two printers.

**Figure 2:**
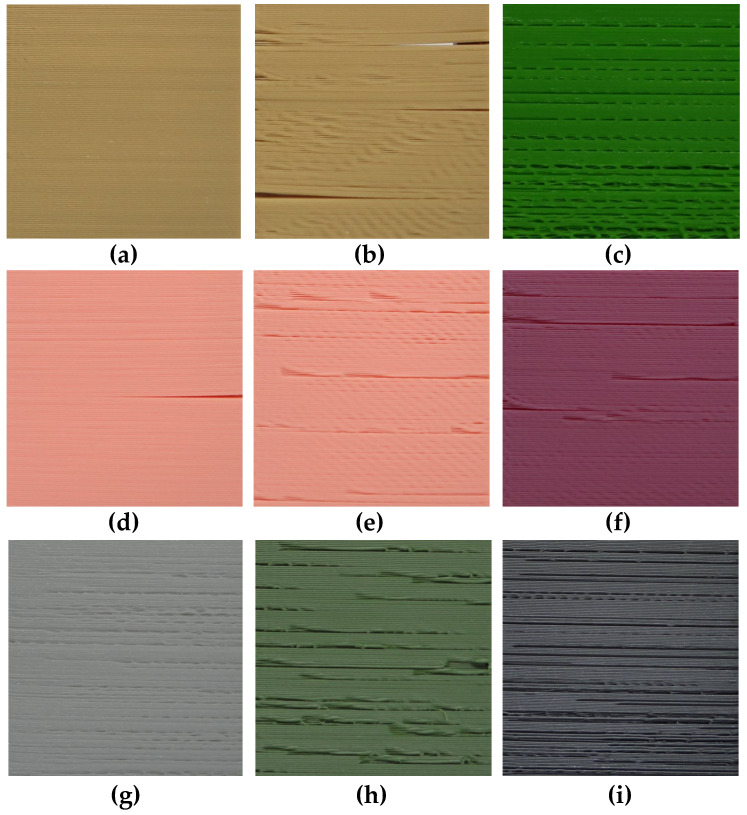
Exemplary photos of 3D printed flat surfaces: high quality sample No. 24 (**a**); and the illustration of various distortions obtained for lower quality 3D prints (**b**–**i**).

Some of the samples were intentionally distorted by forcing “dry printing” and some changes of filament’s delivery speed or temperature as well as parameters of stepper motors. Some of such obtained distortions, including the presence of cracks in some of the ABS samples, are illustrated in Figure 2. The only high quality sample (Figure 2a) is an illustration of properties of the surfaces that were subjectively assessed as representing high quality, whereas the sample in Figure 2d was evaluated as moderately high quality due to the presence of a single crack and minor visible distortions. The remaining seven images are examples of low quality surfaces.

All the manufactured samples were assessed by the members of our team as high, moderately high, moderately low or low quality, independently for each side, and therefore each sample was analyzed and assessed twice (in fact, for most of the samples, the assessment results of both sides were the same). Since the calculations of entropy values for each color channel, as well as using various color to greyscale conversion methods for such obtained images, did not lead to satisfactory results, the decision about the use of 3D scanning technology was made.

The entropy values, considered as statistical measure of randomness, expected to be relatively low for the high quality samples with visible regular patterns, were calculated according to the well known formula:

(1) $$E=-\sum p\cdot \log_{2}p$$

where *p* contains the normalized histogram counts obtained for greyscale images.

The reason for poor classification results based on the entropy analysis of photos was related to the strong dependence of entropy on the color and brightness of the analyzed samples. Although the results obtained for each color separately were in accordance with expectations, the color independent quality assessment of the 3D printed surfaces was troublesome. Another problem, influencing the results, also obtained using the flatbed scanner instead of a camera, as shown in earlier experiments [28], was related to a partial transparency of some of the filaments, particularly visible for some PLA materials.

To ensure the independence of the entropy values on the color of filament, it can be noticed that any distortions of the structure of consecutive layers should also be noticeable in the depth maps which may be obtained using stereovision camera pairs or 3D scanning technology. As laser 3D scanning technology is time-consuming and requires precise control of the moving laser light source, we focused on the use of fringe pattern based 3D scanning.

**Figure 3:**
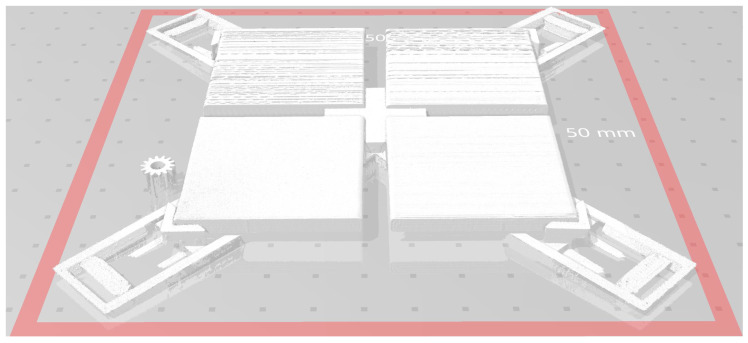
Illustration of the exemplary obtained STL model of four 3D scanned samples with visible mounting elements.

After the first attempts made with the use of previously developed 3D scanning system [25,26], in further experiments the more precise ATOS device manufactured by GOM company was used for the verification of the idea. During the experiments, precise fringe patterns were projected onto the scanned surface of the object and captured by two cameras based on the stereo camera principle allowing to scan the reflective surfaces more precisely (to several micrometers). However, it is worth noticing that projected fringe patterns should not be parallel to the visible layers and therefore the perpendicular patterns were used during the 3D scanning. The obtained cloud of points can be stored in the popular STL format, as shown in Figure 3, illustrating the exemplary scan of four 3D printed samples mounted in the dedicated mounting holders.

**Figure 4:**
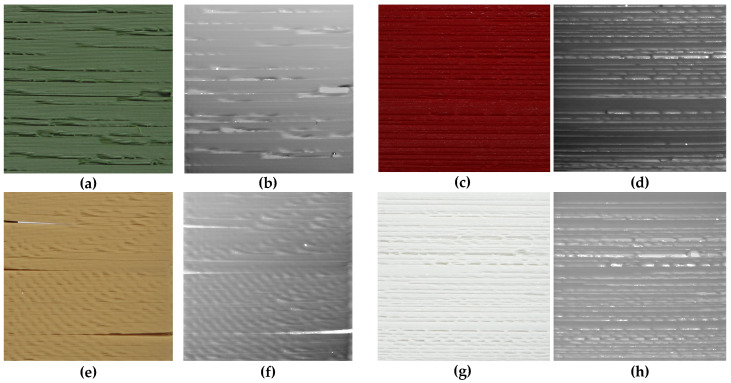
Exemplary lower quality samples: (**a**,**c**,**e**,**g**); and their obtained depth maps: (**b**,**d**,**f**,**h**).

**Figure 5:**
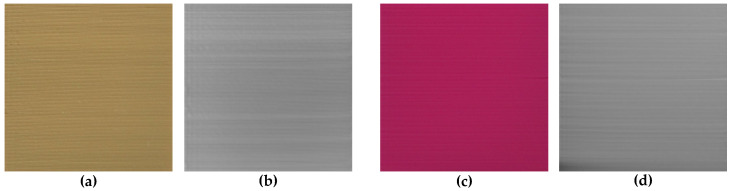
Exemplary high quality samples: (**a**,**c**); and their obtained depth maps: (**b**,**d**).

Such obtained STL files were converted into normalized depth maps stored as 1928×1928 pixels 16-bit greyscale images, representing the surfaces of the 3D printed and scanned samples. Some exemplary depth maps together with photos of respective samples obtained for green, red, brown and white lower quality 3D prints are presented in Figure 4, whereas similar examples obtained for high quality surfaces are illustrated in Figure 5.

The most relevant preprocessing step was the application of the Contrast Limited Adaptive Histogram Equalization (CLAHE) [33] for balancing the distribution of brightness on the images representing the depth maps. These differences, similar to the effect on non-uniform lighting, originate from the placement of the scanned samples that were not always perfectly parallel to each other. Consequently, after scanning four samples at once, as illustrated in Figure 3, some of them may be represented by various depth ranges equivalent to different brightness values of depth map images. Some differences for the opposite corners of the scanned sample can be observed as well. It can also be noticed for both depth maps obtained for the left images shown in Figure 4 (i.e., green and brown samples).

Since the direct application of entropy calculated for the depth maps not always leads to satisfactory results, an approach utilizing the variance of the local entropy values was used, which was previously proposed for images of 3D printed samples obtained using flatbed scanner [34]. Nevertheless, in the previously proposed solution, due to some problems with color independence, the application of the combined metric based on on the average local entropy and its variance was used. Additionally, such a metric has been calculated for the hue component in HSV color space as well as the average of the RGB channels. Finally, the color independent metric was obtained and verified for 18 PLA samples. However, its application for the 126 samples used in our experiments did not lead to satisfactory results.

To overcome the problem of non-uniform brightness of the obtained depth maps, apart from the use of the CLAHE algorithm, the local entropy values as well as their variance were utilized. After extensive experimentation, the best results were obtained assuming the division of the depth map images into 64 blocks forming the array of 8×8 subimages. For each of these regions, the local entropy value $E_{local}$ was calculated in addition to the overall entropy calculated for the whole depth map image, according to Equation (1).

The computations were supplemented with the variance of the local entropy $Var_{E}$ and the final quality metric is proposed as:

(2) $$Q=\log[E_{global}\cdot avg\left(E_{local}\right)\cdot {Var_{E}}^{0.25}]$$

where $avg(E_{local})$ denotes the mean of the 64 local entropy values.

## 4. Results and Discussion

**Figure 6:**
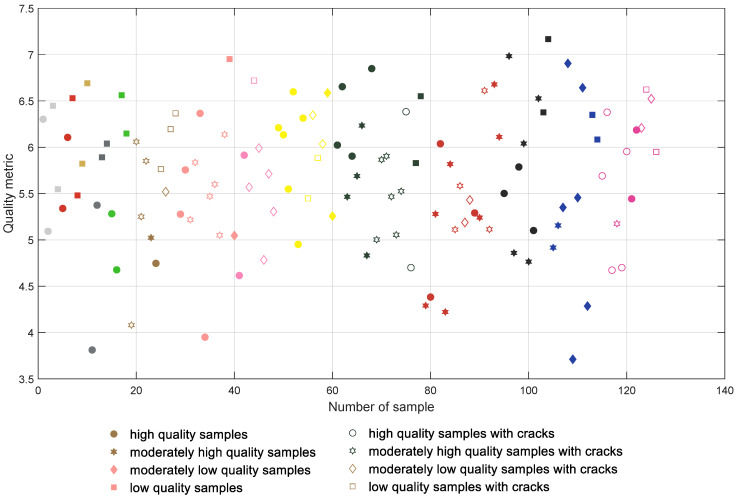
Results obtained for the entropy calculated for depth maps without preprocessing.

**Figure 7:**
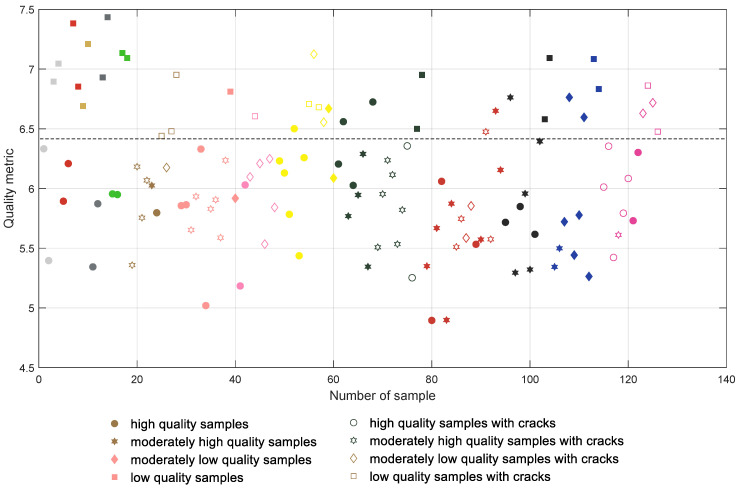
Results obtained for the entropy calculated for depth maps after the CLAHE application.

**Figure 8:**
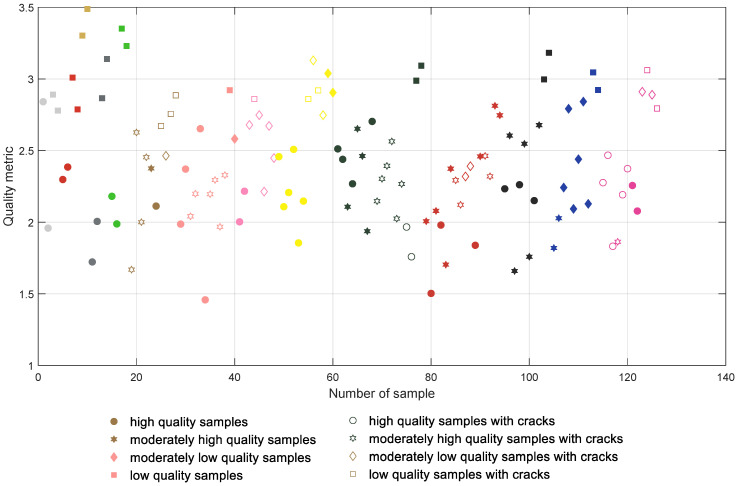
Results obtained for the proposed metric calculated for depth maps without preprocessing.

**Figure 9:**
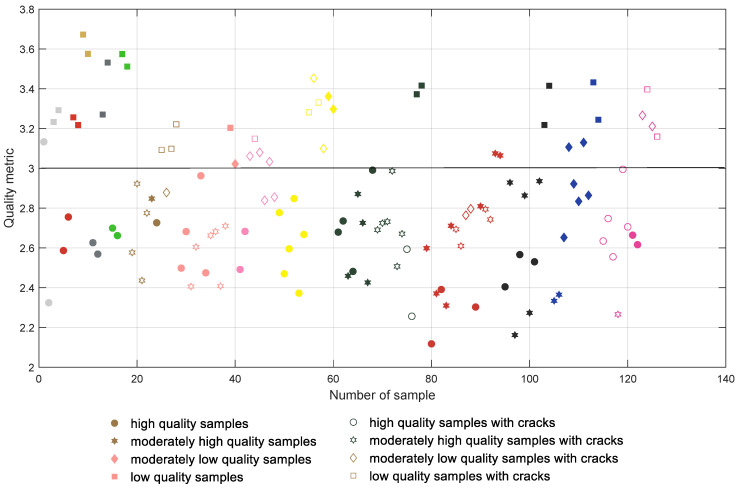
Results obtained for the proposed metric calculated for depth maps after the CLAHE application.

The initial results achieved for the direct computation of the global entropy for the depth map images are presented in Figure 6 where all samples are presented in groups according to their colors and type of filament (with the first 18 samples being manufactured using PLA filaments). The samples containing cracks are marked with unfilled symbols and each color of the mark represents roughly the color of the respective filament. High quality samples are tagged with more round symbols, whereas lower quality ones are marked with more polygonal shapes. The same convention is also used in Figure 7, Figure 8 and Figure 9.

All plots presented in Figure 6, Figure 7, Figure 8 and Figure 9 should be interpreted considering the high values of all presented quality metrics as equivalent to low quality. Since the presence of local distortions causes the increase of entropy as well as its variance, higher values of the metric may be expected, which can be interpreted as a measure of the amount of distortions. Nevertheless, values close to zero might be expected only for perfectly flat surfaces which do not contain any patterns.

Analyzing the distribution of the global entropy values presented in Figure 6, it can be noted that the appropriate classification of the samples according to their quality is impossible. Even for the first four white samples the entropy values are somehow mixed, similar to most of the other colors. A much better situation takes place after the application of the CLAHE algorithm, leading to the results shown in Figure 7 where the separating line can be proposed at 6.4. Nevertheless, some of the samples are still incorrectly classified, e.g., Nos. 1 (white), 26 (brown), and 40 (salmon pink color); many moderately low quality pink samples (Nos. 43–48); two dark green samples; or some of the dark red and blue samples.

**Figure 10:**
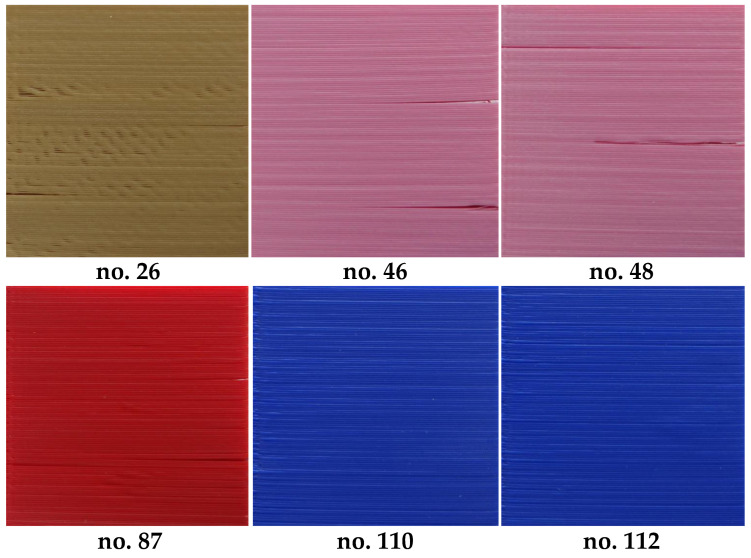
Exemplary moderate quality samples being problematic for the proposed method.

Although the direct application of the proposed metric does not ensure a satisfactory classification, as shown in Figure 8, its use for the depth map images subjected to CLAHE based preprocessing leads to the promising results illustrated in Figure 9. Applying the proposed solution, most of the samples can be correctly classified, although some individual samples seem to be troublesome, i.e., Nos. 1, 26, 46, 48, 87 and 88, as well as four of the blue samples. Nevertheless, incorrect classification takes place mainly for some of the moderately high or moderately low quality samples and in some cases differences between the visual quality of such surfaces are quite small, as illustrated in Figure 10.

To illustrate the advantages of the proposed method numerically, some typical classification metrics were calculated for the proposed method. Assuming the classification of the samples into two classes—high quality (positives) and low quality (negatives)—true positives (TP) can be defined as high and moderately high quality samples classified as “good”. On the other hand, true negatives (TN) are properly classified distorted surfaces (low and moderately low quality). Consequently, false negatives (FN) and false positives (FP) may be assigned to improperly classified samples. Therefore, some of the most popular classification metrics, namely F-Measure (F1-score), specificity, sensitivity and accuracy, may be calculated as:

(3) $$\mathrm{FM}=\frac{2\cdot \mathrm{TP}}{2\cdot \mathrm{TP}+\mathrm{FP}+\mathrm{FN}},$$

(4) $$\mathrm{Specificity}=\frac{\mathrm{TN}}{\mathrm{FP}+\mathrm{TN}},$$

(5) $$\mathrm{Sensitivity}=\frac{\mathrm{TP}}{\mathrm{TP}+\mathrm{FN}},$$

and

(6) $$\mathrm{Accuracy}=\frac{\mathrm{TP}+\mathrm{TN}}{\mathrm{TP}+\mathrm{FP}+\mathrm{FN}+\mathrm{TN}}\;.$$

Their values obtained for the results illustrated in Figure 6, Figure 7, Figure 8 and Figure 9 are shown in Table 1.

The reason of some issues for the proper automatic quality assessment of blue prints is related to the specific type of distortions that are present on their surfaces. Since in these cases the over-extruding of the filament may be observed, resulting in filling the groove between the consecutive layers rather than causing the presence of holes, the expected entropy values should decrease. Therefore, the proposed method cannot be efficiently applied for such specific type of distortions. However, in many applications, the inability to recognize individual layers could be considered as an advantage rather than a problem. An illustration of the surface of one such sample is presented in Figure 10. As can be observed, the visual quality of the samples in the top row is slightly lower than the bottom ones despite their identical subjective assessment.

## 5. Conclusions

The automatic objective quality assessment method of 3D printed surfaces based on the analysis of their depth maps acquired using a fringe patterns based 3D scanner provides encouraging results. The validity and usefulness of the proposed approach was confirmed by the F-Measure exceeding 92% and classification accuracy over 90% for 126 testing samples. In contrast to some machine learning or neural network based solutions which might be potentially used, the presented approach does not require any training process. Potential issues resulting from some imperfections related to therelative position and orientation of the scanned samples according to the 3D scanner can be solved by the application of adaptive histogram equalization using the CLAHE method leading to the correction of the brightness of the depth map images.

The proposed method may be used for further actions such as aborting the printing or correction of the printed surface. The time necessary for printing a single layer—depending on its height—is typically several times longer than the analysis of a single image. Even considering the additional time necessary for 3D scanning using the fringe pattern approach, the total processing time would be short enough to take appropriate action. In practical applications, there is no need to evaluate the quality of the highest layer immediately, especially assuming the location of the camera providing a side view, and therefore some (reasonably short) delays are not critical. In some applications, even without the analysis of the depth maps, the use of a single camera may be useful for monitoring and preventing, e.g., fire caused by heated filament. Nevertheless, these issues have been considered as separate problems, appropriate for further research, and therefore are not analyzed in the paper in details.

Our planned future work will concentrate on the verification of possibilities of data fusion, considering depth maps and photos of the printed surfaces, towards even better classification accuracy. Another direction of our further research is the idea of hybrid quality metrics combining some previously proposed methods.

## Figures and Tables

**Table 1:** The values of classification metrics obtained using the proposed method in comparison to some other approaches presented in the paper.

	Number of Samples				Classification Metrics			
Method	TP	TN	FP	FN	F-Measure	Specificity	Sensitivity	Accuracy
Global Entropy (Figure 6)	65	20	26	15	0.760	0.435	0.813	0.675
Entropy + CLAHE (Figure 7)	74	32	14	6	0.881	0.696	0.925	0.841
Q without CLAHE (Figure 8)	75	36	10	5	0.909	0.783	0.938	0.881
Q with CLAHE (Figure 9)	**77**	**37**	**9**	**3**	**0.928**	**0.804**	**0.963**	**0.905**

## Abbreviations

The following abbreviations are used in this manuscript:

ABS	Acrylonitrile Butadiene Styrene
CAD	Computer Aided Design
CLAHE	Contrast Limited Adaptive Histogram Equalization
CNC	Computerized Numerical Control
FDM	Fused Deposition Modeling
GLCM	Gray-Level Co-occurrence Matrix
IQA	image quality assessment
OCT	Optical Coherence Tomography
SSIM	Structural Similarity
STL	Stereolithography file format
SVM	Support Vector Machines
PLA	Polyactic Acid

## References

[1] Stephens B., Azimi P., Orch Z.E., Ramos T. (2013). Ultrafine particle emissions from desktop 3D printers. Atmos. Environ..

[2] Azimi P., Zhao D., Pouzet C., Crain N.E., Stephens B. (2016). Emissions of Ultrafine Particles and Volatile Organic Compounds from Commercially Available Desktop Three-Dimensional Printers with Multiple Filaments. Environ. Sci. Technol..

[3] Zeltmann S.E., Gupta N., Tsoutsos N.G., Maniatakos M., Rajendran J., Karri R. (2016). Manufacturing and Security Challenges in 3D Printing. JOM.

[4] Busch S.F., Weidenbach M., Fey M., Schäfer F., Probst T., Koch M. (2014). Optical Properties of 3D Printable Plastics in the THz Regime and their Application for 3D Printed THz Optics. J. Infrared Millim. Terahertz Waves.

[5] Straub J. Automated testing and quality assurance of 3D printing/3D printed hardware: Assessment for quality assurance and cybersecurity purposes. Proceedings of the 2016 IEEE AUTOTESTCON.

[6] Fang T., Jafari M.A., Bakhadyrov I., Safari A., Danforth S., Langrana N. Online defect detection in layered manufacturing using process signature. Proceedings of the IEEE International Conference on Systems, Man and Cybernetics.

[7] Fang T., Jafari M.A., Danforth S.C., Safari A. (2003). Signature analysis and defect detection in layered manufacturing of ceramic sensors and actuators. Mach. Vis. Appl..

[8] Cheng Y., Jafari M.A. (2008). Vision-Based Online Process Control in Manufacturing Applications. IEEE Trans. Autom. Sci. Eng..

[9] Szkilnyk G., Hughes K., Surgenor B. Vision Based Fault Detection of Automated Assembly Equipment. Proceedings of the ASME/IEEE International Conference on Mechatronic and Embedded Systems and Applications, Parts A and B.

[10] Chauhan V., Surgenor B. (2015). A Comparative Study of Machine Vision Based Methods for Fault Detection in an Automated Assembly Machine. Procedia Manuf..

[11] Chauhan V., Surgenor B. (2017). Fault detection and classification in automated assembly machines using machine vision. Int. J. Adv. Manuf. Technol..

[12] Laucka A., Andriukaitis D. (2015). Research of the Defects in Anesthetic Masks. Radioengineering.

[13] Fok K.Y., Cheng C., Ganganath N., Iu H., Tse C.K. (2018). An ACO-Based Tool-Path Optimizer for 3D Printing Applications. IEEE Trans. Ind. Inform..

[14] Straub J. (2015). Initial Work on the Characterization of Additive Manufacturing (3D Printing) Using Software Image Analysis. Machines.

[15] Straub J. Alignment issues, correlation techniques and their assessment for a visible light imaging-based 3D printer quality control system. Proceedings of the SPIE Proceedings—Image Sensing Technologies: Materials, Devices, Systems, and Applications III.

[16] Straub J. Identifying positioning-based attacks against 3D printed objects and the 3D printing process. Proceedings of the SPIE—Pattern Recognition and Tracking XXVII.

[17] Straub J. 3D printing cybersecurity: detecting and preventing attacks that seek to weaken a printed object by changing fill level. Proceedings of the SPIE—Dimensional Optical Metrology and Inspection for Practical Applications VI.

[18] Straub J. Physical security and cyber security issues and human error prevention for 3D printed objects: Detecting the use of an incorrect printing material. Proceedings of the SPIE—Dimensional Optical Metrology and Inspection for Practical Applications VI.

[19] Tourloukis G., Stoyanov S., Tilford T., Bailey C. Data driven approach to quality assessment of 3D printed electronic products. Proceedings of the 38th International Spring Seminar on Electronics Technology (ISSE).

[20] Makagonov N.G., Blinova E.M., Bezukladnikov I.I. Development of visual inspection systems for 3D printing. Proceedings of the 2017 IEEE Conference of Russian Young Researchers in Electrical and Electronic Engineering (EIConRus).

[21] Holzmond O., Li X. (2017). In situ real time defect detection of 3D printed parts. Addit. Manuf..

[22] Scime L., Beuth J. (2018). Anomaly detection and classification in a laser powder bed additive manufacturing process using a trained computer vision algorithm. Addit. Manuf..

[23] Delli U., Chang S. (2018). Automated Process Monitoring in 3D Printing Using Supervised Machine Learning. Procedia Manuf..

[24] Sitthi-Amorn P., Ramos J.E., Wangy Y., Kwan J., Lan J., Wang W., Matusik W. (2015). MultiFab: A Machine Vision Assisted Platform for Multi-material 3D Printing. ACM Trans. Graph..

[25] Pajor M., Grudziński M. (2015). Intelligent Machine Tool – Vision Based 3D Scanning System for Positioning of the Workpiece. Solid State Phenom..

[26] Pajor M., Grudziński M., Marchewka Ł. (2018). Stereovision system for motion tracking and position error compensation of loading crane. AIP Conf. Proc..

[27] Okarma K., Fastowicz J. No-Reference Quality Assessment of 3D Prints Based on the GLCM Analysis. Proceedings of the 2016 21st International Conference on Methods and Models in Automation and Robotics (MMAR).

[28] Fastowicz J., Okarma K., Chmielewski L.J., Datta A., Kozera R., Wojciechowski K. (2016). Texture Based Quality Assessment of 3D Prints for Different Lighting Conditions. Proceedings of the Computer Vision and Graphics: International Conference, ICCVG 2016.

[29] Okarma K., Fastowicz J., Tecław M., Chmielewski L.J., Datta A., Kozera R., Wojciechowski K. (2016). Application of Structural Similarity Based Metrics for Quality Assessment of 3D Prints. Proceedings of the Computer Vision and Graphics: International Conference, ICCVG 2016.

[30] Wang Z., Bovik A., Sheikh H., Simoncelli E. (2004). Image quality assessment: From error measurement to Structural Similarity. IEEE Trans. Image Proc..

[31] Fastowicz J., Bąk D., Mazurek P., Okarma K., Choraś M., Choraś R.S. (2018). Estimation of Geometrical Deformations of 3D Prints Using Local Cross-Correlation and Monte Carlo Sampling. Image Processing and Communications Challenges 9: 9th International Conference, IP&C 2017 Bydgoszcz, Poland, September 2017.

[32] Fastowicz J., Okarma K., Silhavy R., Senkerik R., Kominkova Oplatkova Z., Prokopova Z., Silhavy P. (2017). Entropy Based Surface Quality Assessment of 3D Prints. Artificial Intelligence Trends in Intelligent Systems: Proceedings of the 6th Computer Science On-Line Conference 2017 (CSOC2017), Vol 1.

[33] Zuiderveld K., Heckbert P.S. (1994). Contrast Limited Adaptive Histogram Equalization. Graphics Gems IV.

[34] Okarma K., Fastowicz J., Kurzynski M., Wozniak M., Burduk R. (2018). Color Independent Quality Assessment of 3D Printed Surfaces Based on Image Entropy. Proceedings of the 10th International Conference on Computer Recognition Systems CORES 2017.

